# Ageism in cardiac surgery: is less really more?

**DOI:** 10.18632/aging.101701

**Published:** 2019-01-02

**Authors:** Andrew B. Goldstone, Y. Joseph Woo

**Affiliations:** 1 Department of Cardiothoracic Surgery, Stanford University School of Medicine, Stanford, CA 94305, USA

**Keywords:** coronary artery disease, coronary artery bypass grafting, arterial conduits

The adage “less is more” directly applies to many aspects of medicine, and in particular, cardiac surgery. The older an individual patient, the higher a surgeon’s threshold is for performing a more extensive or complicated operation. When it comes to coronary artery bypass grafting (CABG), this may mean leaving concomitant valve regurgitation unrepaired, bypassing fewer vessels, or using different conduits.

Coronary artery disease is the leading cause of death in the United States and Europe, and its prevalence is only expected to increase as the population ages. Thanks to numerous randomized comparison studies [1–3], most specialists would agree that CABG remains the optimal treatment for severe disease. The umbrella term, “CABG,” describes the overall operation, but the manner in which the operation is performed is highly variable. For example, a surgeon may perform the operation with or without cardiopulmonary bypass, or s/he may use arteries instead of veins to bypass diseased coronaries.

Worldwide, the most commonly performed CABG operation bypasses the left anterior descending coronary artery with the left internal thoracic artery (ITA), and the other coronary arteries with saphenous vein. However, the better long-term patency of the left ITA compared with the saphenous vein prompted surgeons to explore other arteries for CABG conduits, namely the right ITA and the radial artery. The right ITA and radial artery are promising conduits; however, until recently, contradictory results from single-center observational studies and concern for early graft failure and sternal wound infection, limit widespread use.

Recently, we conducted a statewide retrospective cohort study of 59,432 patients to compare the effectiveness of second arterial conduits with that of venous conduits for CABG in California [4]. We found that second arterial conduit use is low and declining: <10% of patients received a second arterial conduit in the last year of the study. However, receipt of a second arterial graft was associated with significantly lower mortality (13.1% versus 10.6% at 7 years; hazard ratio [HR], 0.79; 95% confidence interval [CI] 0.72–0.87) and cardiovascular events such as myocardial infarction and repeat revascularization. A right ITA offered no benefit over that of a radial artery, but did increase the risk of sternal wound infection. Notably, an exploratory analysis suggested that second arterial conduits were associated with significantly lower mortality in patients up to 78 years of age at the time of surgery.

Even more recently, Gaudino and colleagues conducted a patient-level meta-analysis of 1,036 patients who participated in randomized, controlled trials comparing radial artery grafts with saphenous vein grafts [5]. The investigators demonstrated that radial artery grafts were associated with a lower incidence of adverse cardiac events compared with saphenous vein grafts (HR 0.67, 95% CI 0.49 to 0.90), and at follow-up angiography, radial artery grafts were associated with a significantly lower risk of occlusion. Despite this, after a mean follow-up of 5 years, the use of radial artery grafts was not associated with a lower incidence of death from any cause compared with saphenous vein grafts. The authors are in the process of collecting follow-up data in this cohort to determine if improved graft patency and lower rates of cardiovascular events will translate into lower mortality in the long term.

Why do surgeons in America avoid using arterial grafts? The vague recommendations that arterial conduits be “considered in appropriate patients” or in those with “reasonable life expectancy” offer little guidance to surgeons [6,7]. In most centers, the rare patient that receives a second arterial conduit is young and with few comorbidities. Our data suggests that individuals nearing 80 years old may benefit from a second arterial conduit. Coupled with the fact that survival after CABG is excellent – 85% to 90% of patients will survive beyond 5 years – it appears that many patients may be clinically appropriate candidates and have “reasonable life expectancy.” Perhaps it’s time surgeons reconsider the notion that “less is more” in elderly patients undergoing cardiac surgery, particularly where arterial versus venous grafts in CABG are concerned.
